# 243. Association Between SARS-CoV-2 Viral Load and Patient Symptoms and Clinical Outcomes Using Droplet Digital PCR

**DOI:** 10.1093/ofid/ofac492.321

**Published:** 2022-12-15

**Authors:** Elizabeth Hastie, Harold Amogan, David Looney, Sanjay R Mehta

**Affiliations:** UCSD, San Diego, California; CFAR Sequencing Core Facility, La Jolla, California; VA San Diego and UCSD, San Diego, California; San Diego VA Medical Center and University of California San Diego, San Diego, California

## Abstract

**Background:**

Droplet digital PCR (ddPCR) has been shown to be more sensitive and precise in the quantification of SARS-CoV-2 when compared to traditional quantitative RT-PCR. Multiple studies have explored associations between SARS-CoV-2 viral load and patient outcomes; however, few have used ddPCR technology. Here we investigated the associations between viral load measured using ddPCR and clinical presentation and outcomes.

**Methods:**

We performed a retrospective observational study of individuals who tested positive for COVID-19 at the VA San Diego between August 2020 and December 2021. SARS-CoV-2 viral load from nasopharyngeal swabs was determined using ddPCR. Baseline demographics, past medical history, clinical course, and laboratory data were abstracted from the chart.

**Results:**

A total of 696 individuals were included, 86% (n=603) of whom were male. The average age was 50-years-old [range: 19-98]. Three-quarters of individuals (76%, n=528) were unvaccinated at diagnosis. Frequency of comorbidities are shown in Table 1. The majority of individuals developed symptoms with 75% (n=516) reporting respiratory symptoms, 47% (n=317) fever, 34% (n=230) GI symptoms, and 23% (n=161) loss of taste and/or smell. A total of 24% of veterans were evaluated only in the emergency department, 21% (n=149) were admitted to the hospital; 9% (n=60) required ICU level of care, 33% of these (n=20) required intubation, and 16 individuals died during hospitalization.

SARS-CoV-2 log_10_ viral load was not associated with age, and only a weak correlation was seen with time from onset of symptoms (r^2^=-0.1, p=0.04). No association was observed between viral load and peak CRP, ferritin, d-dimer, or nadir absolute lymphocyte count. Mean viral load was significantly higher in veterans reporting fever (5.0 vs 5.4, p=0.02) and respiratory symptoms (4.7 vs 5.3, p=0.01). Interestingly, vaccinated veterans also had higher viral loads(5.8 vs 5.0, p< 0.0001).

Baseline characteristics of individuals with COVID-19

**Figure:**
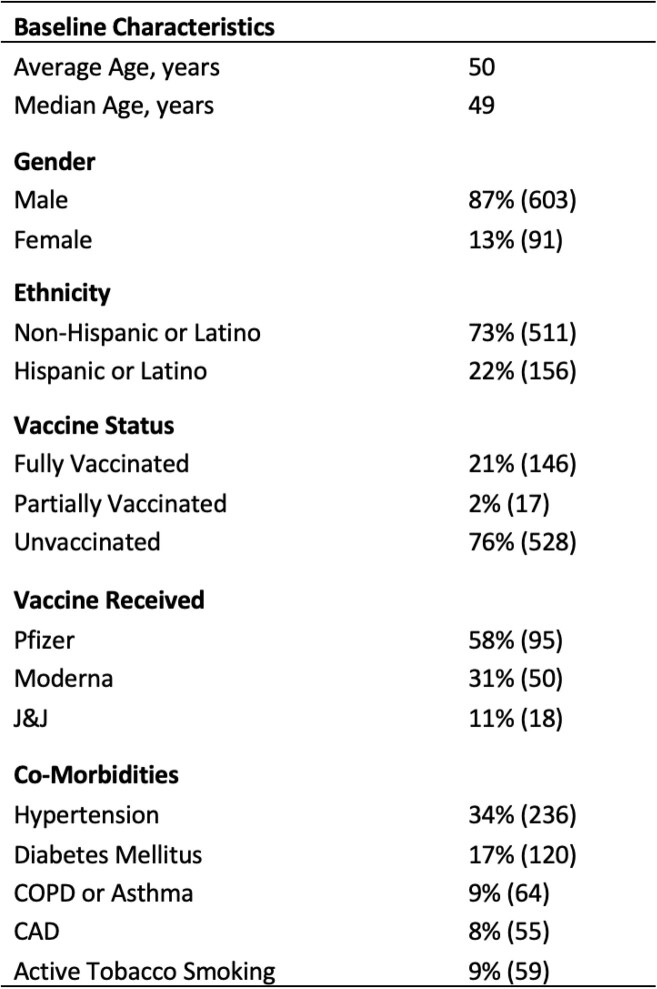

Histogram of COVID-19 RNA viral load

**Figure:**
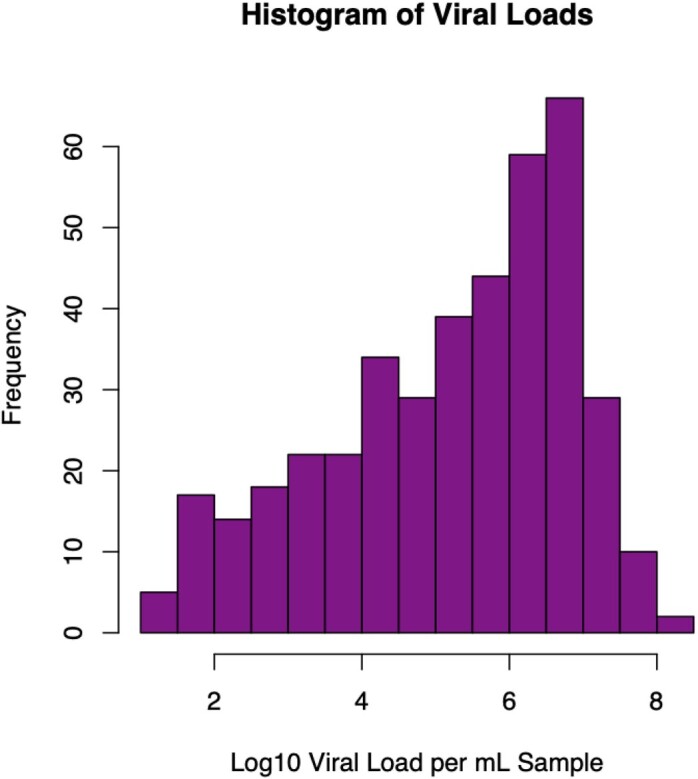

**Conclusion:**

Fever and respiratory symptoms were associated with higher viral loads as expected. The association of vaccination with higher viral load may reflect selection bias for infections in the delta wave. Future work will include multivariate analyses to adjust for medical history and timing of sampling.

**Disclosures:**

**Sanjay R. Mehta, MD**, Zibdy Health: Advisor/Consultant.

